# Corona-Finanzhilfen des Bundes zur Entlastung der Kommunen

**DOI:** 10.1007/s10273-020-2784-2

**Published:** 2020-11-20

**Authors:** Thomas Döring

**Affiliations:** Fachbereich Gesellschaftswissenschaften, Hochschule Darmstadt, Haardtring 100, 64295 Darmstadt, Deutschland

**Keywords:** H70, H79

## Abstract

Die Corona-Krise hat bei den Kommunen zu zusätzlichen Ausgaben und hohen Steuerausfällen geführt. Zur Stabilisierung der Kommunalfinanzen hat der Bund zwei Gesetzesinitiativen auf den Weg gebracht. Neben einer dauerhaft stärkeren Beteiligung des Bundes an den Kosten für Unterkunft und Heizung in der Grundsicherung für Arbeitssuchende sollen einmalig und in pauschaler Form die krisenbedingten Gewerbesteuermindereinnahmen der Kommunen kompensiert werden. Beide Maßnahmen führen jedoch zu einer unsachgerechten Vermischung von kurzfristigen Interventionen zur fiskalischen Bewältigung der Corona-Krise mit grundlegenden Reformnotwendigkeiten der Kommunalfinanzen.

## Abstract (en)

The Corona crisis has led to unforeseeable additional expenses as well as significant losses of tax income for local governments. In order to stabilise public finances at the local level, the German federal government has passed two new bills. One of the bills basically provides for a permanent expansion of the federal government’s financial contribution towards the cost of housing and heating as part of the guaranteed minimum income for job seekers. The federal government also singularly compensates - in cooperation with the state governments - local governments for the significant crisis-related decline in local business tax revenue through a lump sum payment in 2020. However, these two measures will affect short-term intervention to cope with the fiscal problems of the Corona crisis as well as the fundamental need to reform public finances at local government level.

Die Corona-Krise hat einen makroökonomischen Schock ausgelöst, der zur stärksten Rezession in der bundesdeutschen Geschichte geführt hat. Die Bewältigung der Pandemie fordert neben Bund und Ländern auch die Kommunen. Dabei verursachen krisenbedingte Maßnahmen, etwa beim Gesundheitsschutz oder der Unterstützung der lokalen Wirtschaft, zusätzliche Ausgaben. Hinzu kommen hohe Ausfälle bei den Steuereinnahmen, die in erster Linie durch kurzfristige Einnahmenrückgänge bei der Gewerbesteuer sowie dem kommunalen Anteil an der Einkommensteuer bewirkt werden (Freier und Geißler, 2020). Während Bund und Länder die grundgesetzlich verankerte Ausnahmeregelung der Schuldenbremse für eine kreditfinanzierte Ausgabentätigkeit bzw. zur Kompensation von Einnahmeausfällen nutzen können, sind die Kommunen diesbezüglich haushaltsrechtlich deutlich stärker beschränkt. Angesichts der aktuellen Wirtschaftssituation ebenso wie des Haushaltsrechts droht vielen Kommunen daher kurzfristig eine finanzielle Notlage. Dies zu verhindern, fällt entsprechend der bundesdeutschen Föderal- und Finanzverfassung in erster Linie in die Verantwortung der Länder. Angesichts des historischen Ausmaßes der Krise kann auch der Bund zeitlich begrenzt zur Stabilisierung der Kommunalfinanzen beitragen. Er hat zu diesem Zweck zwei Gesetze zur fiskalischen Unterstützung der kommunalen Haushalte verabschiedet (Bundestag, 2020a,b):

- Der Bund beteiligt sich ab sofort dauerhaft mit bis zu 74 % an den kommunalen Ausgaben für die Leistungen für Unterkunft und Heizung in der Grundsicherung für Arbeitssuchende nach § 22 Abs. 1 SGB II. Zur Umsetzung dieser Maßnahme erfolgte eine Änderung der grundgesetzlichen Regelungen in Art. 104a Abs. 3 GG, welche die Bundesbeteiligung an den genannten Ausgaben bislang auf unter 50 % begrenzt, um die Beteiligungsform in Gestalt der Bundesauftragsverwaltung zu vermeiden.
- Der Bund trägt zudem – gemeinsam mit den Ländern – 2020 einmalig und in pauschaler Form zur Kompensation der krisenbedingten Gewerbesteuermindereinnahmen der Kommunen in Höhe von 6,134 Mrd. Euro (gesamter Kompensationsbetrag unter Einbezug des Länderanteils: 10,968 Mrd. Euro) bei. Der Ausgleich soll von den Ländern an die Gemeinden auf Grundlage der erwarteten Gewerbesteuerausfälle weitergeleitet werden. Für die Umsetzung dieser Maßnahme wurde das Grundgesetz durch Art. 104h GG ergänzt, da die unmittelbare Bundeshilfe zugunsten der Kommunen die föderale Zuständigkeitsverteilung unterläuft, der zufolge nur die Länder – nicht aber der Bund – in unmittelbare finanzielle Beziehungen zu den Kommunen treten dürfen.

**Prof. Dr. Thomas Döring** ist Leiter des Zentrums für Forschung und Entwicklung (ZFE) sowie Inhaber der Professur für Politik und Institutionen mit Schwerpunkt Institutionenökonomik an der Hochschule Darmstadt.

Darüber hinaus sollen die Haushalte der ostdeutschen Länder im Zeitraum von 2021 bis 2024 im Umfang von 1,422 Mrd. Euro entlastet werden, um auf diese Weise finanzielle Spielräume zur Stärkung der kommunalen Investitionen zu generieren. Zu diesem Zweck hat der Bund seinen Anteil an den Erstattungen für die Aufwendungen der Rentenversicherung aus den Zusatzversorgungssystemen der ehemaligen DDR von bislang 40 % auf 50 % erhöht. Im Gegenzug sinkt der von den Ländern im Beitrittsgebiet zu tragende Anteil von 60 % auf nur noch 50 %.[1]

### Erhöhte Beteiligung an den Kosten für Unterkunft

Angesichts der negativen fiskalischen Folgewirkung der COVID-19-Pandemie auf die Haushaltssituation der Kommunen ist eine finanzielle Unterstützung von Städten und Gemeinden in der aktuellen Ausnahmesituation generell zu begrüßen, um die Erfüllung von pflichtigen wie freiwilligen Selbstverwaltungsaufgaben im Bereich von kommunaler Daseinsvorsorge und Infrastrukturbereitstellung zu gewährleisten. Aus Sicht der föderalen Finanzverfassung sollten hierbei jedoch Landes- vor Bundeshilfen den Vorrang haben. Eine Beteiligung des Bundes an den Mehrausgaben der Kommunen sollte im Gegenzug eine absolute Ausnahme bleiben. Hierfür spricht auch die Erwartung, dass auf Länderebene noch vor der Bundesebene mit einem Erreichen des Vor-Corona-Niveaus bei den Steuereinnahmen gerechnet werden kann, was unter anderem auf die vom Bund für diverse Länderaufgaben zusätzlich abgegebenen Umsatzsteueranteile zurückzuführen ist. Dass der Bund nun die Kommunen gerade bei den Leistungen für Unterkunft und Heizung in der Grundsicherung für Arbeitssuchende (KdU) unterstützt, ist zwar insofern nicht überraschend, als die kommunalen Mehrbelastungen krisenbedingt vornehmlich bei den Ausgaben für Gesundheit und Soziales – davon geschätzt 2 Mrd. Euro allein gemäß SGB II (Henneke, 2020) – anfallen. Als problematisch kann dennoch bewertet werden, dass die erhöhte KdU-Beteiligung – anders als im Fall der Gewerbesteuermindereinnahmen – nicht einmalig, sondern dauerhaft und damit über den krisenbedingten Anlass hinaus erfolgen soll.

Für eine differenzierte Bewertung der genannten Maßnahme sind zudem die folgenden finanzwissenschaftlichen Erkenntnisse von grundlegender Bedeutung (Döring, 2001):

- Um die aus allokativen Gründen ökonomisch wünschenswerte politische Selbst- und Eigenverantwortung nachgeordneter Staatsebenen (hier: Länder und Kommunen) zu stärken, wird aus Sicht des Fiskalföderalismus allgemein gefordert, dass die Zuordnung von Ausgabenkompetenzen an der Verteilung der Aufgabenkompetenzen orientiert ist. So ist eine parlamentarische Kontrolle nur gegenüber einer Verwaltung möglich, die für die Erfüllung der Aufgaben und die dafür erforderlichen Ausgaben gleichermaßen verantwortlich ist. Die Ausrichtung der Ausgaben an der Aufgabenkompetenz wird aber auch mit der Erfahrung begründet, dass nur im Fall einer vertikalen Kongruenz von Aufgaben- und Ausgabenverteilung jene „Kräfte zum Zuge [kommen], die den Erfordernissen einer geordneten und wirtschaftlichen Haushaltsführung Geltung zu schaffen suchen“ (Fischer-Menshausen, 1980). Eine diesen Überlegungen folgende Ausgabenzuordnung wird bekanntermaßen mit der Realisierung des Prinzips der Konnexität gleichgesetzt.

- Die Realisierung des Konnexitätsprinzips gilt allerdings in den Fällen als problematisch, in denen die verschiedenen Teilkompetenzen einer staatlichen Aufgabe nicht ungeteilt sind, was vor allem dann gilt, wenn Entscheidungs- und Vollzugskompetenzen auseinanderfallen. Welcher der beiden Aufgabenkompetenzen die Ausgabenzuständigkeit folgen soll, ist finanzwissenschaftlich allerdings umstritten (Wust, 1981; Geske, 1998; Döring, 2004). So wird die Orientierung an der Vollzugskompetenz gefordert, wenn die Länder (und ihre Kommunen) für die Gesetzesdurchführung verantwortlich sind. In Deutschland stellt dies den Regelfall dar (Art. 83 GG in Verbindung mit Art. 104a Abs. 1 GG). Die meisten Ökonomen betrachten demgegenüber eine Ausrichtung an der Entscheidungskompetenz als angemessen (wer bestellt, soll auch bezahlen). Eine Zuordnung der Ausgabenkompetenz kann aus Sicht des Fiskalföderalismus jedoch nur dann eindeutig erfolgen, wenn die Entscheidungs- und Vollzugskompetenzen selbst nicht von mehreren Ebenen wahrgenommen werden.

- Solche Mischkompetenzen können insbesondere zwei Ausprägungen haben: 1. Die gemeinschaftliche Aufgabenwahrnehmung durch mehrere Gebietskörperschaftsebenen (Bund, Länder, Kommunen) sowie 2. die Rahmensetzung einer übergeordneten Staatsebene (Bund) für Tätigkeiten der nachgeordneten Gebietskörperschaften (Länder und Kommunen). Der hier zu betrachtende Fall der erhöhten Kostenübernahme durch den Bund bei Leistungen für Unterkunft und Heizung im Rahmen der grundgesetzlichen Regelungen zu den Geldleistungsgesetzen (Art. 104a Abs. 3 GG) fällt in die zweite Kategorie. Die damit einhergehende Rahmensetzung ist insofern als problematisch anzusehen, als diese dazu führen kann, dass übergeordnete Staatsebenen die Aufgabenerfüllung von Umfang und Struktur her weitgehend determinieren bei gleichzeitiger Externalisierung der damit verbundenen Finanzierungslasten.

1 Zudem hat der Bund bereits im Rahmen seines Konjunkturpakets eine finanzielle Unterstützung für die Corona-bedingten Ausfälle beim ÖPNV zur Entlastung der Kommunen gewährt.

- Werden die aus einer Aufgabe und den damit verbundenen Ausgaben resultierenden Finanzierungslasten nicht einer Ebene allein zugewiesen, kommt es – so auch bei den Leistungen für Unterkunft und Heizung – zu sogenannten Mischfinanzierungen in der Aufgabenerfüllung. Solche Mischfinanzierungen bergen allerdings die Gefahr, Ausgabenentscheidungen zu verzerren, da die politischen Akteure einer jeden Gebietskörperschaftsebene dem gesamten Nutzen einer öffentlichen Aufgabenerfüllung nicht die gesamten Kosten, sondern lediglich die eigenen Finanzierungsbeiträge gegenüberstellen. Zugleich enthalten solche Mischfinanzierungen eine Tendenz „zu undurchschaubaren Entscheidungs- und Koordinierungsbürokratismen, die einen Anstieg der Verwaltungskosten bedeuten" (Bösinger, 1995). In Anbetracht dessen wurden in der Vergangenheit wiederholt unter anderen auch die grundgesetzlichen Regelungen zu den Geldleistungsgesetzen (Art. 104a Abs. 3 GG) kritisiert, da sie von einer dem Konnexitätsprinzip entsprechenden Zuordnung der Ausgabenverantwortung abweichen.

Bewertet man vor dem Hintergrund dieser grundlegenden finanzwissenschaftlichen Überlegungen die geplante dauerhaft erhöhte Übernahme der Leistungen für Unterkunft und Heizung durch den Bund, ergeben sich die folgenden Schlussfolgerungen: Einerseits kann der um 25 % auf dann 74 % angestiegene Finanzierungsanteil des Bundes an den KdU gemäß SGB II insofern als gesteigerte Realisierung des Konnexitätsprinzip im Sinne einer Orientierung an der Gesetzgebungskompetenz eingestuft werden, wie dies den Entscheidungsbefugnissen des Bundes im genannten Aufgabenbereich besser als bislang Rechnung trägt.

Positiv zu bewerten ist zudem, dass es zu keiner vollständigen Übernahme der Kosten von Leistungen für Unterkunft und Heizung kommt, da eine vollständige Erstattung aller mit dem Gesetzesvollzug verbundenen Kosten durch den Bund zu Fehlanreizen führen würde. Dies gilt insofern, als die Kosten der Aufgabenerfüllung in diesem Bereich nicht allein durch die gesetzliche Regelung der Aufgabe, sondern ebenso durch den Vollzug bestimmt sind (Oebbecke, 2020; Döring und Stahl, 2000). Da Ländern und Kommunen im Gesetzesvollzug durchführungsbezogene Ermessensspielräume offenstehen, würde eine Garantie der vollständigen Kostenübernahme zu ineffizienten Ergebnissen führen. Dies betrifft nicht allein die Verwaltungsausgaben, sondern ebenso die Zweckausgaben, da Länder und Kommunen unter einer Konnexitätsregel im Sinne der Gesetzeskausalität weder daran interessiert sein dürften, die Kosten je Einzelfall der Inanspruchnahme von KdU-Leistungen noch die Zahl der Fälle insgesamt in Grenzen zu halten. Unter der Zielsetzung einer effizienten Ausgabenpolitik wäre eine über die 75 %-Grenze hinausgehende Kostenübernahme daher wenig zweckdienlich.

### Beibehaltung der bestehenden Mischfinanzierung

Andererseits ändert sich durch die Steigerung des Bundesanteils an den KdU nichts am Tatbestand, dass es sich hierbei auch weiterhin um eine Form der Mischfinanzierung mit Folgewirkungen in Gestalt einer potenziell ebenso ineffizienten wie mit (parlamentarischen) Kontrollproblemen versehenen Aufgabenerfüllung handelt. Es kann daher kritisiert werden, dass die zur Steigerung des Bundesanteils an den KdU vollzogene Verfassungsänderung, womit die grundgesetzlich geregelten Voraussetzungen der Bundesauftragsverwaltung modifiziert werden, als „verfassungssystematisch falsch" einzustufen ist (Bundesrechnungshof, 2020). Die bislang diesbezüglich geltende Verfassungsregel sah vor, dass in Fällen der Bundesauftragsverwaltung (Art 85 GG), zu denen bislang auch Geldleistungsgesetze mit einer Bundesbeteiligung von 50 % und mehr zählten, sowohl der Bund allgemeine Verwaltungsvorschriften erlassen kann als auch die Landesbehörden (einschließlich der involvierten Kommunen) den Weisungen des Bundes unterstehen. Zwar handeln die Länder auch im Rahmen der Auftragsverwaltung in eigener Verantwortung, sie unterliegen jedoch der uneingeschränkten Bundesaufsicht. Die Aufsicht bezieht sich dabei auf die Gesetzmäßigkeit ebenso wie die Zweckmäßigkeit der Aufgabenausführung (einschließlich eines Prüfrechts gemäß § 91 BHO durch den Bundesrechnungshof).

Unter den bisherigen Regelungen der Bundesauftragsverwaltung hätte im Fall einer gestiegenen Bundesbeteiligung besser kontrolliert werden können, inwieweit die Kommunen – wie in der Vergangenheit wiederholt geschehen – als Aufgabenträger vor Ort dem Bund tendenziell zu hohe Beträge für die KdU in Rechnung stellen. Auch erleichtert eine Aufgabenerfüllung im Rahmen der Bundesauftragsverwaltung der Bundesebene die parlamentarische Kontrolle der Mittelverwendung, da sie im Unterschied zur Lastverteilungsregel gemäß Art. 104 Abs. 3 GG für eine stärkere institutionelle Kongruenz in der Verantwortung von Aufgabenerfüllung und den dafür zu tätigenden Ausgaben sorgen würde, wie dies aus Sicht des Fiskalföderalismus grundsätzlich gefordert wird. Aus diesem Blickwinkel ist die Änderung des Grundgesetzes als nicht notwendig zu bewerten.

Gegen eine Bundesauftragsverwaltung bei diesen Geldleistungen, bei der die Länder fachlich wie finanziell der Bundesaufsicht unterstehen würden, könnte allerdings sprechen, dass davon auszugehen ist, das Länder und Kommunen gerade im Sozialbereich im Unterschied zum Bund über bessere Kenntnisse der arbeitsmarkt- und sozialpolitisch relevanten Gegebenheiten vor Ort verfügen und insofern eine lokal adäquatere Falleinschätzung und Maßnahmenplanung gewährleisten können. Im Einklang mit dem Subsidiaritätsgrundsatz, demzufolge eine untere Staatsebene nur bei systematischer Überforderung durch eine höhere Ebene unterstützt werden sollte, ist daher aus finanzwissenschaftlicher Sicht auch eine weitgehende Dezentralisierung der Finanzierungsverantwortung im Fall der KdU prinzipiell vorstellbar, was wiederum dem Konzept der Vollzugskausalität nach 104a Abs. 1 GG entspricht. Der Unterschied zu einer Konnexitätsregel im Sinne der Gesetzeskausalität ist darin zu sehen, dass die aufgrund der Sozialgesetzgebungskompetenz des Bundes auch in diesem Fall erforderliche Kompensationszahlung des Bundes nicht variabel in Orientierung an den tatsächlichen KdU, sondern pauschaliert zu erfolgen hätte (z.B. über einen höheren Umsatzsteueranteil von Ländern und Kommunen oder höheren Bundeszuweisungen im Rahmen des Länderfinanzausgleichs). Mit solchen Pauschaltransfers wird das bestehende Anreizproblem einer übermäßigen Ausdehnung der Kosten von vornherein ausgeschlossen (Huber und Lichtblau, 1999).

Für eine solche stärker auf Länderebene verankerte Aufgaben- und Ausgabenkompetenz würde zudem sprechen, dass dadurch verhindert werden könnte, dass sich die Länder ihrer Finanzierungsverantwortung gegenüber den Kommunen entziehen, wie dies in der Vergangenheit bisweilen dann der Fall war, wenn Bundesmittel zur Unterstützung von kommunalen Aufgaben gewährt wurden. Die sogenannten Regionalisierungsmittel des Bundes, die Bundeshilfen im Rahmen des Gemeindeverkehrsfinanzierungsgesetzes oder auch die Krankenhausfinanzierung haben gezeigt, dass entsprechende finanzielle Unterstützungen des Bundes nicht vollumfänglich von den Ländern an die Kommunen weitergegeben wurden oder die Länder entsprechende Bundeshilfen dazu genutzt haben, um sich selbst nicht länger adäquat an der Erfüllung kommunaler Aufgaben zu beteiligen.

## Ausgleich der Gewerbesteuerausfälle

Nicht allein mit Blick auf die Aufgabenerfüllung und die damit verbundene Ausgabentätigkeit der Kommunen, sondern auch bezogen auf die Einnahmeseite der kommunalen Haushalte sind hinsichtlich der Folgewirkungen der Covid-19-Pandemie finanzielle Hilfen generell gerechtfertigt. Von den wichtigsten Finanzierungsquellen der Kommunen, zu denen neben den Steuern (2019: 104,4 Mrd. Euro) ebenso Zuweisungen (2019: 100,7 Mrd. Euro) und Gebühren (2019: 33,6 Mrd. Euro) zählen, sind alle drei genannten Einnahmearten von der Wirtschaftskrise betroffen (Statistisches Bundesamt, 2020). Richtet man den Blick allein auf die kommunalen Steuereinnahmen, nehmen hier sowohl die Gewerbesteuer (2019: 42,6 Mrd. Euro nach Abzug der Umlagen an Bund und Land) als auch der Anteil der Kommunen an der Einkommensteuer (2019: 39,7 Mrd. Euro) eine zentrale Stellung ein. Anders als die Grundsteuer sowie der kommunale Umsatzsteueranteil, die beide wenig konjunkturreagibel sind, korreliert das Aufkommen der Gewerbesteuer ebenso wie jenes aus dem Anteil an der Einkommensteuer direkt mit der wirtschaftlichen Entwicklung. Wenn nun der Bund in dieser Situation – und nicht allein die Länder, wie dies die föderale Finanzordnung nahelegen würde – durch entsprechende Finanzhilfen zur Stabilisierung der kommunalen Finanzlage beiträgt, kann dies aus ökonomischer Sicht insofern als gerechtfertigt gelten, als der Bund im Rahmen seiner infektions- und seuchenschutzpolitischen Kompetenzen durch den verhängten Lockdown die aktuelle finanzielle Schieflage der Kommunen maßgeblich mit verursacht hat.

Jenseits dessen sollte allerdings auch klar sein, dass krisenbedingt nicht allein mit Mindereinnahmen bei der Gewerbesteuer, sondern ebenso mit nennenswerten Einnahmeausfällen der Kommunen bei der Einkommensteuer gerechnet werden muss, für die bislang jedoch keine fiskalische Kompensation vorgesehen ist. Zwar dürften die Mindereinnahmen bei der Einkommensteuer – nicht zuletzt aufgrund der „Sockelgrenze" (Zimmermann und Döring, 2019) – weit weniger ausgeprägt als bei der Gewerbesteuer sein. Nichtsdestotrotz reagiert die Einkommensteuer auf Verwerfungen am Arbeitsmarkt, was die Kommunen mit ihrem Anteil an der Einkommensteuer am allgemeinen Steuerrückgang beteiligt (Freier und Geißler, 2020). Weit dramatischer dürfte jedoch der Einbruch bei der Gewerbesteuer (netto) ausfallen, bei der – je nach Annahme bezüglich des zu erwartenden Rückgangs des Bruttoinlandsproduktes – mit Steuerausfällen zwischen 5,8 Mrd. Euro und 28,7 Mrd. Euro gerechnet wird. Der weit überwiegende Teil der Gewerbesteuermindereinnahmen fällt dabei bei den Kommunen in Westdeutschland an. Der Grund für die dramatischen Gewerbesteuerausfälle ist auf deren Bemessungsgrundlage – im Wesentlichen die Gewinne der Unternehmen – zurückzuführen. Eine wirtschaftliche Krise im aktuellen Ausmaß führt bei den meisten Unternehmen zu erheblichen Gewinneinbußen bzw. zu Verlusten. Dies bedingt eine hohe Volatilität der Gewerbesteuer. Darüber hinaus kann sich in einer einzelnen Kommune die Situation gänzlich anders – mithin deutlich dramatischer – darstellen. Dabei gilt allgemein: Je höher der Finanzierungsanteil der Gewerbesteuer an den gesamten kommunalen Einnahmen ist, desto größer fällt der Einnahmenverlust in der gegenwärtigen Rezession aus.

Anders als in der Stellungnahme des Bundesrates (2020) zum Gesetzentwurf zur Änderung des Grundgesetzes (Art. 104a und 143h) vom 3. Juli 2020 gefordert, wird eine Streichung des Passus „auf Grundlage der erwarteten Mindereinnahmen" als Orientierungsmaßstab für die Verteilung der Ausgleichszahlungen von Bund und Ländern zur Kompensation der Gewerbesteuermindereinnahmen als nicht erforderlich angesehen. Begründet wurde die Forderung nach Streichung damit, dass die im Gesetzentwurf genannte Verteilungsregel so „ausgelegt werden [kann], dass die erwarteten Mindereinnahmen jeder einzelnen Kommune zugrunde gelegt werden müssen“, was weder als leistbar noch als belastbar eingeschätzt wird. Demgegenüber ist jedoch damit zu rechnen, dass für die von den Ländern zu verteilenden Mittel die durchschnittlichen Einnahmen der zurückliegenden Jahre, die über die Finanzstatistik vorliegen, einen naheliegenden Maßstab bilden werden. Zudem sollten die gewährten Kompensationsmittel als eigene Einnahmen der Kommunen verbucht werden, um so zur Stabilisierung der kommunalen Finanzausgleiche beizutragen.

## Grundlegende Gewerbesteuerreform erforderlich

Der notwendig gewordene Ausgleich der Gewerbesteuermindereinnahmen der Kommunen verweist im Kern auf ein tieferliegendes Problem, eine nach wie vor ausstehende grundlegende Reform der Gewerbesteuer bzw. des kommunalen Einnahmensystems insgesamt. Für eine solche strukturelle Reform sind aus finanzwissenschaftlicher Sicht die folgenden Kriterien relevant (Döring und Feld, 2005):

- Im Mittelpunkt steht das Ziel einer Verstetigung der kommunalen Einnahmen, d. h. konjunkturelle Schwankungen sollten nicht so stark auf die kommunalen Einnahmen durchschlagen, wie dies aktuell krisenbedingt vor allem bei der Gewerbesteuer der Fall ist. Eine stabile und bedarfsgerechte Einnahmenerzielung ist dabei insofern bedeutsam, als die überwiegende Zahl der kommunalen Ausgaben (Personalausgaben, Infrastruktur, Gebäudeunterhaltung etc.) unabhängig von kurzfristigen konjunkturellen Schwankungen anfällt. Im Bereich der kommunalen Sozialleistungen verändert sich der Ausgabebedarf sogar antizyklisch, d. h. in der Rezession steigen die finanziellen Belastungen.

- Die Kommunen sollten über eine oder mehrere vom Aufkommen her nennenswerte Steuerquelle(n) mit Hebesatzrecht verfügen, wie dies Art. 28 Abs. 2 Satz 3 GG zur Sicherung der kommunalen Selbstverwaltung normiert. Aus ökonomischer Sicht kann damit dem Grundsatz der fiskalischen Äquivalenz Rechnung getragen werden, dessen Erfüllung eine an den Präferenzen der ortsansässigen Wohnbevölkerung und Unternehmen orientierte Aufgabenerfüllung gewährleistet.[2]

- Als weitere Zielsetzung wird eine möglichst geringe interkommunale Streuung des Steueraufkommens gefordert. Es handelt sich dabei um eine das spezifisch deutsche Verständnis von Föderalismus kennzeichnende Forderung, die im grundgesetzlichen Postulat der Wahrung gleichwertiger Lebensverhältnisse (Art. 72 Abs. 2 GG) seinen Niederschlag gefunden hat. Auf die kommunale Ebene angewendet besagt dieser Grundsatz, dass die Einnahmenausstattung zwischen den Kommunen nicht zu stark differieren soll. Dies auch deshalb, damit auf umfangreiche korrigierende Ausgleichszahlungen über den Finanzausgleich verzichtet werden kann.

- Schließlich ist eine Substanzbesteuerung zu vermeiden. Unternehmen sollen durch die Besteuerung nicht zur Aufgabe ihres Geschäftsbetriebs gezwungen werden und daher nur Steuern zahlen, wenn sie auch Gewinne erzielen. Aufgrund einer Substanzbesteuerung würden Unternehmen aus dem Markt ausscheiden, die sich ohne diese Form der Besteuerung dort halten könnten. Aus diesem Grund wurden in früheren Reformen der Gemeindefinanzen bereits die Lohnsummensteuer und die Gewerbekapitalsteuer abgeschafft.

Vor allem der im letztgenannten Punkt bereits enthaltene Verweis auf die Gewerbesteuer und die Beschneidung von deren Bemessungsgrundlage über die Zeit auf eine mittlerweile reine Ertrags- bzw. Gewinnsteuer erklärt deren extreme Konjunkturreagibilität, die in dieser Ausgestaltung keinen Beitrag zur Verstetigung der kommunalen Einnahmen leisten kann. Auch fällt die interkommunale Streuung des Gewerbesteueraufkommens – bundesländerübergreifend und infolge einer räumlich unterschiedlichen Verteilung von Wirtschafts- und Steuerkraft – vergleichsweise groß aus. Zwar gewährleistet die Gewerbesteuer durch das bestehende Hebesatzrecht die Verwirklichung kommunaler Finanzautonomie. Dem Grundsatz der fiskalischen Äquivalenz wird aber nur begrenzt Rechnung getragen, vor allem weil in der Vergangenheit die Bemessungsgrundlage der Gewerbesteuer stetig beschnitten wurde. Dies findet seinen Ausdruck in zahlreichen Befreiungen von der Steuerpflicht (ausgenommen sind Land- und Forstwirtschaft, freie Berufe, Wohnungswirtschaft und öffentliche Betriebe) sowie in immer wieder angehobenen Freibeträgen (für Einzelunternehmen und Personengesellschaften) und einer 1992 eingeführten, progressiv wirkenden Messzahlenstaffel. Die Gewerbesteuer ist damit über die Zeit zu einer Steuer auf mittlere und große Unternehmen (vornehmlich Kapitalgesellschaften) degeneriert (Fuest und Huber, 2003). Zudem hängt das Gewerbesteueraufkommen selbst größerer Städte von nur wenigen Unternehmen ab, wobei Insolvenzen oder unternehmenspolitische Entscheidungen von Jahr zu Jahr zu schmerzlichen Steuerausfällen führen können.

2 Der Wissenschaftliche Beirat beim Bundesministerium der Finanzen (1982) hat in diesem Zusammenhang den Begriff des Interessenausgleichs geprägt. Er besagt, dass die spezifischen Bedarfe einer sozialen Gruppe (Wohnbevölkerung, Unternehmen) innerhalb einer Kommune durch das Aufkommen aus Abgaben finanziert werden sollen, die von den Mitgliedern der gleichen Gruppe aufgebracht werden.

## Kein Mangel an Reformvorschlägen

Zu berücksichtigen ist schließlich auch, dass der Gewerbesteuer mit der Steuerreform 2000 im System der Unternehmensbesteuerung eine völlig neue Rolle zugewiesen wurde. Während für Kapitalgesellschaften durch eine Senkung des Körperschaftsteuersatzes das allgemeine Belastungsniveau der Ertragsteuern niedrig gehalten wurde, konnte eine vergleichbare Entlastung der einkommensteuerpflichtigen Einzelunternehmen und Personengesellschaften nur durch eine pauschale Anrechnung der Gewerbesteuer auf die Einkommensteuer realisiert werden. Dies hat jedoch zur Folge, dass die Entlastung der Personenunternehmen unter anderem von den weiteren Einkommensteuerpflichtigen, also auch von den Arbeitnehmern, die Lohnsteuer zahlen, mitgetragen wird, für die – bei gleichem Einkommensteueraufkommen – die Steuerbelastung ohne Gewerbesteueranrechnung niedriger ausfallen würde. Im Ergebnis wird mit dieser partiellen Weiterwälzung der Gewerbesteuer der Grundsatz der fiskalischen Äquivalenz nicht mehr gewährleistet. Die in der aktuellen Krise entstandenen Mindereinnahmen bei der Gewerbesteuer können insofern auch als das Ergebnis eines spezifischen Politikversagens bei der Ausgestaltung dieser Steuer in den zurückliegenden Jahrzehnten verstanden werden.

In Anbetracht der genannten Defizite der bestehenden Gewerbesteuer kann die Feststellung nicht überraschen, dass nach wie vor deren Reform hohe politische Priorität haben sollte. Neben dem kurzfristigen Ausgleich coronabedingt entstandener Mindereinnahmen wäre mit vergleichbarem Nachdruck auf die Realisierung eines nachhaltigen Gemeindefinanzierungssystems hinzuwirken, um für mehr Resilienz der Kommunalfinanzen im Fall von zukünftigen wirtschaftlichen Krisensituationen zu sorgen. Mit Blick auf die Gewerbesteuer wurden viele Reformvorschläge bereits in der ersten Hälfte der 2000er Jahre vorgelegt, ohne dass irgendeiner dieser Vorschläge bislang politisch umgesetzt wurde. An Aktualität mangelt es all diesen Reformmodellen nicht. Einer groben Einteilung folgend kann dabei zwischen wertschöpfungsorientierten Modernisierungsmodellen einerseits sowie einkommensorientierten Zuschlagsmodellen andererseits unterschieden werden (Broer, 2004; Henckel, 2004). Zu den wertschöpfungsorientierten Reformvorschlägen zählen – ohne Anspruch auf Vollständigkeit – Modelle wie etwa jenes der Bundesvereinigung der kommunalen Spitzenverbände (2003), das im Kern auf eine „Revitalisierung" der Gewerbesteuer hinausläuft (auch Scherf, 2002; Oberhauser, 2003; Färber, Fugmann-Heesing und Junkernheinrich, 2003; Jarass und Obermair, 2003). Zentrale Elemente dieses Vorschlags sind die Erweiterung des Kreises der Steuerpflichtigen auf alle Produktions- und Dienstleistungsbetriebe sowie die Verbreiterung der Bemessungsgrundlage in Richtung der gesamten Nettowertschöpfung bei gleichzeitig massiver Senkung der bisherigen Steuermesszahlen.

Als Beispiel für den zweiten Modelltyp kann der Reformvorschlag des Bundesverbands der Deutschen Industrie und des Verbands der Chemischen Industrie (2001) gelten, der auf eine Abschaffung der bestehenden Gewerbesteuer und deren Ersatz durch einen kommunalen Zuschlag zur Körperschaftsteuer[3] sowie den Ersatz des Gemeindeanteils an der Einkommensteuer durch eine – ebenfalls durch Einführung eines Zuschlagsrechts bewirkte – direkte Einkommensbesteuerung der Bürgerinnen und Bürger durch ihre Kommunen abzielt (Schemmel, 2002; Fuest und Thöne, 2003; Jachmann, 2003; Döring, 2007). Ohne diese Modelle im Detail vorstellen oder gar bewerten zu wollen, verdeutlicht bereits die Fülle an Reformvorschlägen, dass gerade mit Blick auf die Gewerbesteuer – aber auch das kommunale Einnahmensystem insgesamt – ein grundlegender Handlungsbedarf besteht, der durch eine Kompensation der Gewerbesteuermindereinnahmen der Kommunen zum aktuellen Zeitpunkt lediglich kurzfristig abgemildert, aber keineswegs strukturell behoben wird.

## Fazit

Unabhängig von der zurückliegenden Beurteilung der betrachteten Finanzhilfen für die Kommunen ist zunächst grundsätzlich positiv zu bewerten, dass der Bund bereit ist, umfangreiche Mittel zur Milderung der krisenbedingten Finanzierungslasten der Kommunen im Sozialbereich sowie zum Ausgleich der kommunalen Gewerbesteuermindereinnahmen einzusetzen, anstatt diese etwa zur Beteiligung an einem Fonds zur Tilgung kommunaler Altschulden zu verwenden (Junkernheinrich, 2020). Die Lösung des Altschuldenproblems sollte selbst unter dem Eindruck der aktuellen Krisensituation im Sinne des Subsidiaritätsprinzips und seiner Anwendung auf die föderalen Finanzbeziehungen vorrangig Sache der Kommunen sowie – wenn diese nachweislich mit der Lösung des Problems überfordert sein sollten – gegebenenfalls der Länder sein. Eine Beteiligung des Bundes ist demgegenüber nicht zwingend erforderlich. Jenseits dessen gilt für Finanzhilfen des Bundes an die Kommunen, dass diese unter den gegebenen Rahmenbedingungen dann als ökonomisch gerechtfertigt angesehen werden können, wenn sie in klarem Bezug zur momentanen Krisensituation stehen und damit lediglich einmalig bzw. zeitlich befristet gewährt werden. Demgegenüber sollte eine unsachgerechte Vermischung von kurzfristigen Interventionen zur fiskalischen Bewältigung der Corona-Krise mit grundlegenden Reformnotwendigkeiten der föderalen Finanzordnung vermieden werden.

3 Alternativ ist auch eine Betriebssteuer mit Zuschlagsrecht vorgeschlagen worden (Kronberger Kreis, 2003).

Insofern bleibt anzumahnen, dass beides – sowohl die dauerhaft höhere Beteiligung des Bundes an den finanziellen Lasten der Leistungen für Unterkunft und Heizung in der Grundsicherung für Arbeitssuchende als auch der einmalige Ausgleich der krisenbedingten Gewerbesteuermindereinnahmen – lediglich Ad-hoc-Maßnahmen darstellen, welche die nach wie vor aus finanzwissenschaftlicher Sicht anstehenden Reformen bei den Bund-Länder-Finanzbeziehungen ebenso wie beim Gemeindefinanzierungssystem nicht ersetzen können.
